# Tissue Rotation of the *Xenopus* Anterior–Posterior Neural Axis Reveals Profound but Transient Plasticity at the Mid-Gastrula Stage

**DOI:** 10.3390/jdb10030038

**Published:** 2022-09-10

**Authors:** Lyuba Bolkhovitinov, Bryan T. Weselman, Gladys A. Shaw, Chen Dong, Janhavi Giribhattanavar, Margaret S. Saha

**Affiliations:** 1 Department of Molecular Biology, Massachusetts General Hospital, Harvard University, Boston, MA 02114, USA; 2 School of Medicine, Georgetown University, Washington, DC 20007, USA; 3 Department of Anatomy and Neurobiology, Virginia Commonwealth University School of Medicine, Richmond, VA 23298, USA; 4 Department of Developmental Biology, Washington University School of Medicine, St. Louis, MO 63110, USA; 5 Baystate Medical Center, Springfield, MA 01199, USA; 6 Department of Biology, College of William and Mary, Williamsburg, VA 23185, USA

**Keywords:** Xenopus, plasticity, anterior–posterior, axis formation, neural, gastrula, embryo

## Abstract

The establishment of anterior–posterior (AP) regional identity is an essential step in the appropriate development of the vertebrate central nervous system. An important aspect of AP neural axis formation is the inherent plasticity that allows developing cells to respond to and recover from the various perturbations that embryos continually face during the course of development. While the mechanisms governing the regionalization of the nervous system have been extensively studied, relatively less is known about the nature and limits of early neural plasticity of the anterior–posterior neural axis. This study aims to characterize the degree of neural axis plasticity in *Xenopus laevis* by investigating the response of embryos to a 180-degree rotation of their AP neural axis during gastrula stages by assessing the expression of regional marker genes using in situ hybridization. Our results reveal the presence of a narrow window of time between the mid- and late gastrula stage, during which embryos are able undergo significant recovery following a 180-degree rotation of their neural axis and eventually express appropriate regional marker genes including *Otx*, *Engrailed*, and *Krox*. By the late gastrula stage, embryos show misregulation of regional marker genes following neural axis rotation, suggesting that this profound axial plasticity is a transient phenomenon that is lost by late gastrula stages.

## 1. Introduction

Understanding the molecular and cellular mechanisms that govern the formation of the vertebrate embryonic nervous system has been a longstanding goal of developmental biology. Because of intrinsic interest in the nervous system, as well as the implications for both biomedicine and evolutionary biology, neural development has attracted a significant amount of research effort since the beginning of experimental embryology [1,2,3]. This effort has led to considerable progress in defining both the tissue interactions and the cellular and molecular genetic mechanisms mediating the normal development of the nervous systems [3,4,5,6,7,8,9,10,11,12,13,14,15]. However, the development of functional organ systems not only requires the determinative processes that lead to cell type specificity and appropriate patterning of those cell types, but also the ability to maintain this differentiated, patterned state in the face of ongoing genetic and environmental perturbations that occur throughout embryogenesis [16,17,18]. The ability to repair and/or compensate for potentially deleterious alterations in development is often referred to as plasticity or regulative ability. While all cells possess some degree of plasticity in order to respond and adapt to changing, often adverse conditions, pronounced plasticity of fate is a key feature of embryogenesis, one that is required to ensure normal development [19,20,21]. Plasticity is a phenomenon that is particularly prominent in the embryonic nervous system and is especially notable in one of the most prominent features of the nervous system, namely the regionalization along the anterior–posterior axis. This study focuses on plasticity of fate, that is, the ability of cells and tissues to undergo a dramatic change in the phenotype to which they were originally fated.

Often associated with attempts to delineate timing of regional determination and differentiation in the developing nervous system, the plasticity of the AP neural axis has historically been investigated by transplantation experiments in which tissues were excised from one location and placed into an ectopic region or were replaced following rotation. The degree of recovery by the embryos later in development provides information on the nature of such plasticity (reviewed in [22]). Early AP neural axis reversal experiments were performed by Hans Spemann [23,24], who rotated portions of the anterior neural plate and underlying mesoderm of neural plate stage newt embryos. The rotated tissue maintained its previous identity and developed according to its prior position in the embryo, although the result was partially attributed to the vertical signaling from rotated mesoderm [25]. Subsequent transplantation experiments of rotated neural ectoderm tissue at the neural plate stage exhibited conflicting results, with some transplants showing complete recovery [26,27] and others developing in reverse orientation [25]. These inconsistencies were later attributed to a lack of host–donor labeling, leading to potential mesoderm contamination and varying sizes of rotated tissues in different experiments, with larger transplants being less likely to undergo a full recovery [28]. A plethora of other experiments transplanted pieces of tissue into ectopic locations, with equally mixed results. Transplantation of *Xenopus laevis* prospective spinal neuroectoderm tissue to presumptive eye and prosencephalic regions at the neural plate stage resulted in a mixture of anterior and posterior features at the transplant regions, indicating that the patterning of the AP axis is relatively fixed by the neural plate stage [29,30]. Despite inconsistent results due to a lack of molecular assays, these earlier transplantation experiments demonstrated that at the early to mid-gastrula stage, the (AP) patterning of presumptive neuroectoderm is not yet fully determined [30,31].

While these studies suggested a window of AP neural axis plasticity between the early gastrula stage and the neural plate stage, many lacked unambiguous host–donor marking and relied on histology and cell shape to distinguish the regional identities of cells along the AP axis. More recent studies on plasticity have employed molecular markers for host–donor marking as well as cell type identification, but current research on AP neural axis plasticity tends to focus on much smaller regions of tissue along the axis later in development, particularly the hindbrain and neural crest, rather than that of large regions of neuroectoderm [32,33,34,35,36,37,38,39,40,41,42,43].

In order to assess the plasticity of a large swath of presumptive neural tissue, we examined the ability of *Xenopus laevis* to re-pattern a significant portion of the AP neural axis by performing a series of transplants with rotated and non-rotated tissue at mid-gastrula and gastrula stages. Here, we show that *Xenopus laevis* embryos have a narrow, but profound, window of AP neural axis plasticity between mid- and late gastrula stages that allow embryos to repattern regional gene expression and undergo significant, although not perfect, recovery from transplantation of rotated tissues.

## 2. Materials and Methods

### 2.1. Animal Care and Embryo Collection

Because of the easy accessibility of its embryos, the availability of molecular tools, and its emerging importance as a model for regeneration and developmental diseases, *Xenopus laevis* was selected as the model of choice for these experiments [44,45,46,47,48,49]. Material available at Xenbase (http://www.xenbase.org/) accessed on 1 July 2022 also supported the choice of this model system. All animal care and use procedures were conducted in accordance with and approval of the William and Mary Institutional Animal Care and Use Committee, (IACUC) following the 3Rs guidelines for replacement, reduction, and refinement. Protocols, described below, were based upon information in the Xenopus Cold Spring Harbor Protocols (http://cshprotocols.cshlp.org/cgi/collection/xenopus accessed on 1 July 2022) and additional sources, as noted. Adult animals were housed in a designated vivarium aquarium room on 12:12 light/dark cycles in flow through tanks and fed frog chow ad libitum. *Xenopus laevis* embryos were obtained from natural mating following injection of males with 400U Human Chorionic Gonadotropin (HCG) and females with 700 U HCG. Jelly coats were removed from the embryos using a 2% L-cysteine solution (pH 8.0) in 0.1X Marc’s Modified Ringers (MMR), thoroughly rinsed, and placed in 0.1X MMR with 50 μg/mL gentamicin. After sorting to remove any necrotic or improperly dividing embryos, the solution was changed to fresh 0.1X MMR with 50 μg/mL gentamicin. Healthy dividing embryos were bilaterally injected with 4.6 nl 10% fluorescent-linked dextran (FLDx), according to [50,51,52]. Injected embryos were raised in 0.1X MMR with 4% ficoll until gastrula stages, when embryos were screened for strong, uniform fluorescence expression and then transferred to 0.1X MMR. Embryos were staged according to Nieuwkoop and Faber [53].

### 2.2. Neural Ectoderm Transplantation

**Figure 1:**
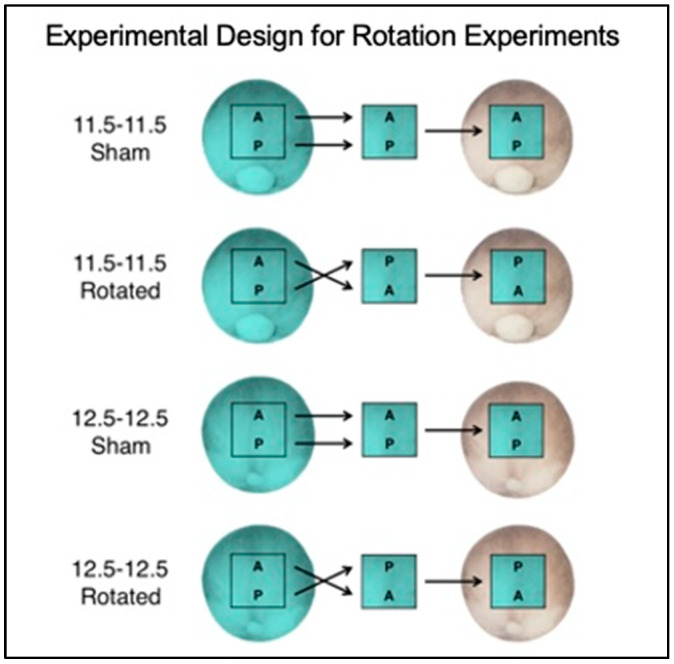
Schematic of experimental design for experiments. Fluorescein-injected donor embryo is on the left and uninjected host embryo is on the right. Dorsal view, with anterior up and posterior down. The precise tissue removed was determined from preliminary fate mapping experiments and published work [54].

For all transplantation surgeries, FlDx-injected embryos were used as donors and uninjected embryos used as hosts in order to delineate donor tissue from host tissue during subsequent analysis. Four different types of surgeries were performed as follows (Figure 1). The neural ectoderm from the medial 50% of the anterior–posterior axis was removed from a mid-gastrula embryo (St. 11.5), rotated 180 degrees, and placed into a host embryo from which an identical piece of the presumptive neural tissue was removed. These will be referred to as mid-gastrula rotated embryos. A sham control experiment was also performed, except the donor tissue was not rotated; these will be referred to as mid-gastrula sham embryos. Additionally, the same experiment was performed at the late gastrula stage (St. 12.5). Here, for late gastrula rotated embryos, the neural ectoderm from the medial 50% of the anterior–posterior axis was removed from a late gastrula embryo (St. 12.5), rotated 180 degrees, and placed into a host embryo from which an identical piece of the presumptive neural tissue was removed. A similar control experiment was performed for the late gastrula transplant experiments, except the tissue was not rotated in late gastrula sham embryos.

The transplantation surgeries were performed as follows. Embryos were transferred to a clay-bottomed dish with 1/3X MMR with 4% ficoll and the vitelline membrane was removed with Dumont No. 5 fine forceps. The embryos were placed snugly into wells indented into the bottom of the clay dish using dull forceps. Embryos were positioned with their dorsal side facing up, anterior pointing away from the experimenter and posterior pointing towards the experimenter. Dissections were performed using Dumont No. 5 fine forceps and needles pulled from 20 μL Corning glass disposable micro-sampling pipets using a Narishige model PB-7 vertical needle puller. First, an incision was made with the needle on the posterior side of the presumptive neural ectoderm, parallel to and approximately 1/4 mm above the blastopore. This initial incision was made at a depth such that it cut through the entire layer of neural ectoderm without damaging the underlying layer of mesoderm. Then, perpendicular cuts were made using the fine forceps to cut out a flap of neural ectoderm approximately 50% of the width of the embryo. The flap was carefully peeled back using the fine forceps. Care was taken to ensure that the underlying mesoderm was not damaged, and that the piece of neural ectoderm had no mesoderm contamination. The explant was snipped off on the anterior side using fine forceps, parallel to the first incision.

The explant of neural ectoderm was first removed from the uninjected host embryo and discarded into solution. Then, the same procedure was performed on the fluorescently labeled donor embryo, but the explant was held using fine forceps and transplanted onto the open space on the dorsal side of the host embryo. During this transplantation, the explant was placed onto the host so that the explant’s original AP axis orientation matched that of the host (Sham transplant) or was rotated 180° relative to the host’s axis (Rotated transplant). This setup resulted in four transplant conditions: 11.5-11.5 Sham, 11.5-11.5 Rotated, 12.5-12.5 Sham, and 12.5-12.5 Rotated (Figure 1).

**Figure 2:**
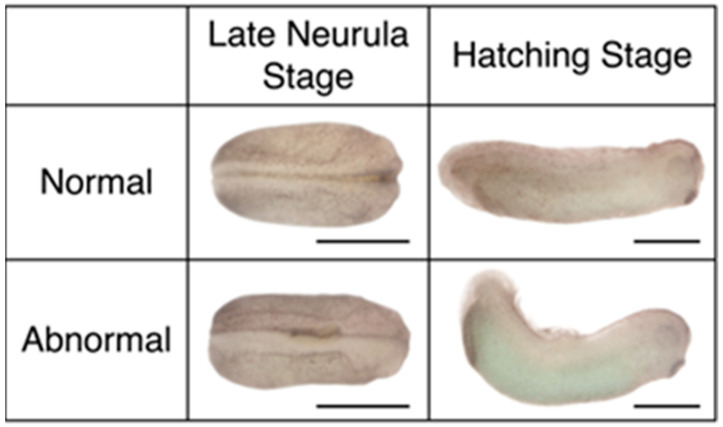
Examples of normal versus abnormal embryos. Significant deviations from embryos shown in Nieuwkoop and Faber [53] were regarded as abnormal. Scale bars = 1 mm.

A small glass chip made from a microscope slide coverslip was positioned to hold the transplant in place to facilitate incorporation. 2–3 h after transplantation, the glass chip was removed, and the embryos were transferred to 0.1X MMR with 4% ficoll. Embryos were allowed to grow to early neurula (stage 14), mid-neurula (stage 15, 16), late neurula (stage 18) or hatching (stage 30) stages, and then were imaged for both bright field and fluorescence using an Olympus SZH10 microscope with an Olympus DP71 camera or a Nikon SMZ800N microscope with a Nikon DS-Ri2 camera. The gross morphology of each embryo was observed and categorized as either normal or abnormal. Embryos classified as abnormal had underdeveloped or malformed neural features, a bent spinal cord, or a shortened body axis (Figure 2). After imaging, embryos were fixed in 1X MEMFA (MOPS/EGTA/Magnesium Sulfate/Formaldehyde Buffer) for subsequent analysis.

Differences in the proportion of embryos among treatment groups that were normal/abnormal/did not survive were analyzed using a chi-square test for the association between experimental treatment and morphology. The Bonferroni correction was used to correct the significance for multiple comparisons, and significance was determined at the *p* < 0.05 level. Statistical tests were run using GraphPad Prism, San Diego, CA, USA, Version 9.1.0.

### 2.3. In Situ Hybridization, FlDx Detection, and Whole Mount Imaging

**Figure 3:**
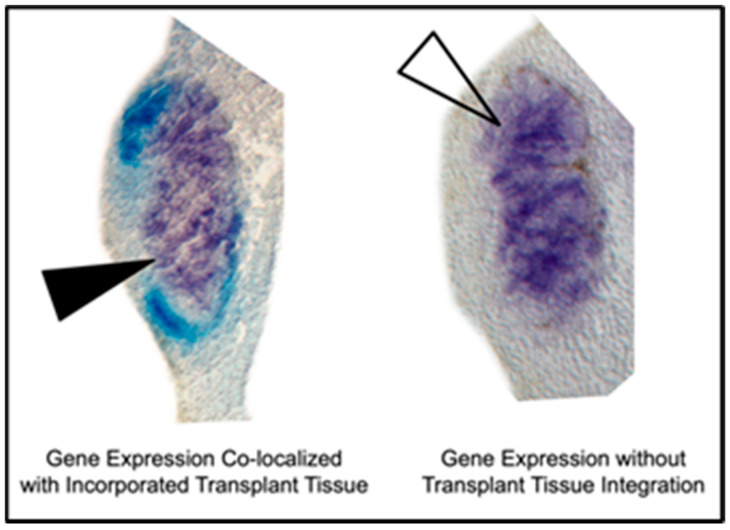
Images showing criteria for discerning between co-localization of gene expression with integrated transplant tissue and endogenous expression. Light blue coloring indicates transplanted tissue and purple coloring indicates *Otx2* expression via in situ hybridization. These specific examples are sections from the mid-gastrula sham surgery showing *Otx2* expression (**left**) and the late gastrula sham surgery showing *Otx2* expression (**right**).

Whole-mount in situ hybridization was performed as described in [55,56] for the regional marker genes *XCG-1* (cement gland), *Otx2* (forebrain and eyes), *En-2* (midbrain- hindbrain boundary), and *Krox20* (rhombomeres 3 and 5 of the hindbrain). Each marker gene was analyzed in a separate embryo. The first color reaction for the gene of interest was performed with NBT/BCIP (nitro blue tetrazolium /5-bromo-4-chloro-3-indolyl-phosphate), resulting in a purple stain. After completion of the first color reaction, embryos were incubated in an anti-fluorescein alkaline phosphatase antibody to mark the location of the fluorescein-injected transplanted tissue, given that using only fluorescence itself was not as sensitive or unambiguous for discrimination between host and donor tissue. The second color reaction for the transplanted tissue was done with BCIP, resulting in an easily distinguishable light blue stain (Figure 3).

To control for variations in assay conditions and standardize signal strength, the time of fixation was determined by the strength and specificity of the gene marker signal in the control embryos (embryos that did not undergo any type of manipulation). After development of signals, embryos were transferred to Bouin’s fixative and fixed overnight at 4 °C. Following fixation, embryos were transferred to bleaching solution and nutated under a fluorescent light to remove pigmentation. After bleaching was completed, embryos were transferred to 1X PBS for whole mount imaging. Embryos were photographed for whole mount photography using either an Olympus SZH10 microscope with an Olympus DP71 camera, or a Nikon SMZ800N microscope with a Nikon DS-Ri2 camera. Photographs were taken at 3X to 5.6X magnification. Whole images were globally (never partially) adjusted for color, brightness, and contrast using Adobe Photoshop CS3, San Jose, CA, USA.

### 2.4. Histology and Imaging of Slides

Following in situ hybridization, embryos were dehydrated by four 15-min washes in ethanol and 1X PBS (first with 75% 1X PBS/25% ethanol, second with 50% 1X PBS/50% ethanol, third with 25% 1X PBS/75% ethanol, and fourth with 100% ethanol). This was followed by three 15-min xylene washes (first with 50% ethanol/50% xylene, second with 100% xylene, and third with 50% xylene/50% paraffin) then two two-hour paraffin incubations. Embryos were positioned in embedding boats filled with paraffin, and the paraffin was allowed to harden at room temperature for approximately 24 h. Embryos were sectioned on a microtome into 20 μm-thick transverse sections, then coverslipped and mounted on microscope slides using FluorMount. They were imaged using an Olympus MU100 camera with AmScope Imaging software. Bright field images were taken at 10X magnification and globally (never regionally) adjusted for brightness and color using Adobe Photoshop CS3.

### 2.5. Histological Gene Expression Analysis

All gene expression analysis was performed on histological sections. Embryos were scored in two categories: “Old Off” and “New On”. “New On” refers to the extent of correct marker gene expression co-localized with transplanted neural tissue based on its new position. Co-localization was assessed using three criteria: (1) if the transplant was contiguous with the host gene expression (2) if the host gene expression was flanked by two areas of transplant on the same side (3) if the transplant was directly dorsal to the gene expression on the same side.

“New On” assesses the degree to which new, appropriate gene expression is turned on in the transplanted tissue based on its new location. Scores were assigned qualitatively from 0–3 to represent the spatial extent of correct gene expression in the embryo. A score of 3 indicates that the “New On” gene was expressed appropriately, covering between 76–100% of the expected, normal expression domain; a score of 2 indicates that the “New On” gene was expressed in 51–75% of the normal expression domain with only 0–25% of the correct spatial expression, and scores of 1, and 0 indicate that “New On” gene was expressed as 26–50% and 0–25%, respectively, of the normal expression domain. In cases where the location of endogenous gene expression did not overlap with transplant incorporation, New On could also be scored as n/a, because the transplanted tissue did not directly express the gene of interest. In some cases, although the region of transplanted tissue did not overlap with the region of expression for the marker gene of interest, the host tissue was still able to bring up correct expression. “Old Off” refers to the ability of an embryo to keep gene expression turned off in areas where the expected gene should not be expressed and is also scored on a qualitative scale of 0 to 3. A score of 0 indicates that the gene is not expressed in ectopic, unexpected areas while a score of 3 indicates that the gene is expressed significantly in neural areas where it should not be expressed. Intermediate scores follow the same percentage guidelines as for New On. All analyses were performed double blind by at least two independent analysts and discrepancies resolved by a third person. If discrepancies could not be resolved, the data was discarded as too ambiguous to include (less than 5% of all embryos).

Regional marker gene expression was assessed for embryos raised to the early, mid-and late neurula stages, as well as the hatching stage. A two-way ANOVA was performed to analyze the effect of transplant stage (mid-gastrula vs. late gastrula) and transplant type (sham vs. rotated) on combined marker gene expression as well as on each marker gene separately. Post hoc analysis was performed using the Tukey post hoc test. All statistical tests were run using GraphPad Prism, Version 9.1.0.

## 3. Results

### 3.1. Experimental Overview

In order to investigate the plasticity of the developing AP neural axis, a series of four neural transplantation experiments were performed (Figure 1). The neural ectoderm from the medial half of the anterior–posterior axis was removed from a mid-gastrula embryo (St. 11.5), rotated 180 degrees, and placed into a host embryo from which an identical piece of the presumptive neural tissue was removed. An identical control experiment was performed, except the tissue was not rotated. The same experiment was performed at the late gastrula stage (St. 12.5). For all experiments, embryos were grown to either the late neurula or hatching stage, and then were assayed for the expression of four regional marker genes expressed along the AP neural axis (*XCG-1, Otx2, En-2,* and *Krox20*).

### 3.2. Mid-Gastrula Rotated Embryos Display Relatively Normal Morphology while Late Gastrula Rotated Embryos Show Significant Abnormality

**Figure 4:**
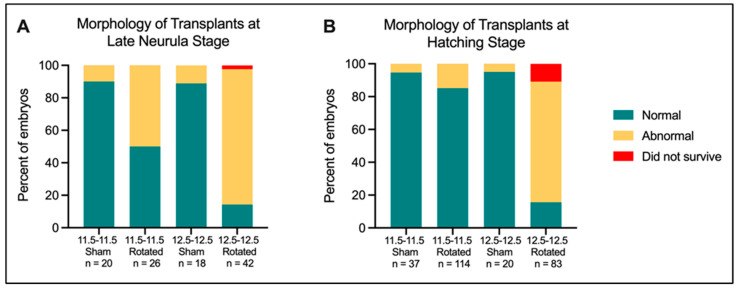
Morphology of embryos with transplanted tissues at late neurula (St. 18) (**A**) and hatching stages (St. 30) (**B**).

In order to determine the overall viability and level of morphological impairment of the embryos following neural ectoderm transplantation, we analyzed the gross morphology of embryos grown up to the late neurula and hatching stages (Figure 4). At the late neurula stage, a chi-square test of independence revealed that morphology (normal vs. abnormal/did not survive) was significantly associated with transplant type (χ2 = 45.12, 3 df, *p* < 0.0001). Sham embryos from transplants performed at both mid-gastrula and late gastrula stages develop normally almost 90% of the time. They both have significantly higher proportions of normally developing embryos than the population distribution (*p* = 0.0009 for the mid-gastrula sham; *p* = 0.0035 for the late gastrula sham). About 50% of mid-gastrula rotated embryos develop normally, compared to only 15% of late gastrula rotated embryos. The proportion of abnormally developing late gastrula rotated embryos is highly significantly different from the population distribution (*p* = 0.0001).

When analyzed at the hatching stage, a chi-square test of independence revealed that morphology (normal vs. abnormal/did not survive) was still significantly associated with transplant type (χ2 = 130.4, 3 df, *p* < 0.0001). Sham transplants showed an even higher proportion of normal development than at the late neurula stage, with over 90% of mid-gastrula sham embryos developing normally. Mid-gastrula rotated embryos showed increasingly normal development at the hatching stage, with around 85% of embryos developing normally. All three of these groups had significantly higher proportions of normal development than the population distribution (*p* = 0.0004 for 11.5-11.5 Sham; *p* = < 0.0001 for 11.5-11.5 Rotated; *p* = 0.0364 for 12.5-12.5 Sham). In contrast, late gastrula rotated embryos had a highly significant increase in the proportion of abnormally developing embryos (*p* = < 0.0001). These results suggest that there is not a loss in overall healing ability as embryos develop from mid-gastrula to late gastrula, because sham transplants at both stages largely develop normally. When performed at mid-gastrula stages, embryos are able to recover on a gross morphological level following neural axis rotation, indicating that the transplanted neural tissue is able to integrate and adapt morphologically to the fate of its new host environment. In contrast, embryos are not able to recover following neural axis rotation at late gastrula stages (St. 12.5), indicating a loss in plasticity of the neural axis.

### 3.3. Mid-Gastrula Rotated Embryos Display Comparatively Normal Regional Marker Gene Expression Compared to Aberrant, Ectopic Gene Expression in Late Gastrula Rotated Embryos

#### 3.3.1. Overview

In order to investigate the plasticity of the AP axis at a molecular level, in situ hybridization was performed for four regional marker genes (*XCG-1*, *Otx2*, *En-2*, and *Krox20*) in mid-gastrula sham and rotated, and late gastrula sham and rotated embryos. Histological sections of each embryo were scored for two categories: “New On”, “Old Off”. As described in Materials and Methods, “New On” refers to the ability of the transplanted tissue to correctly express marker gene in its new location in the host embryo; with a score of 0 indicating 0–25% of correct expression, and scores of 1, 2, and 3 represent up to 50%, 75%, or 100% of correct expression, respectively. A score of “n/a” was given if the transplanted tissue did not incorporate in the location of endogenous gene expression for the particular marker gene. Depending on the stage and marker gene, “n/a” scores ranged from 0% (*Krox,* St. 30) to 47% (*XCG*, St. 18). “Old Off” refers to the ability of the transplanted tissue to suppress (now ectopic) gene expression in the region where it would have been expressed if not transplanted. A score of 0 indicates that no ectopic gene expression was co-localized with transplanted tissue, while a score of 3 indicates large amounts of aberrant gene expression co-localized with the transplant. An embryo with perfect regulation of its AP axis would therefore have a score of 3 for “New On” and a score of 0 for “Old Off.”

#### 3.3.2. Transplants Assayed at Late Neurula Stages

**Figure 5:**
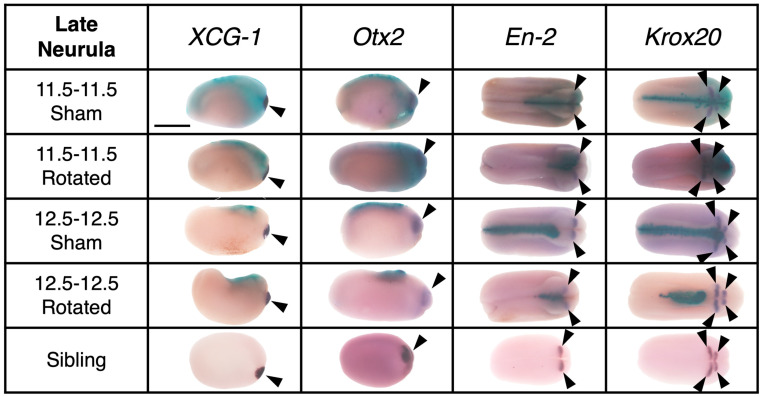
Representative examples of expression of regional marker genes in transplants and sibling controls at late neurula stage (St. 18). With a lateral view with dorsal facing up for *XCG-1* and *Otx2*, and dorsal view for *En-2* and *Krox20*, anterior is to the right in all images. Purple stain marked with a solid arrowhead indicates gene expression and aqua blue stain was used to indicate transplant incorporation. Scale bar represents 1 mm.

**Figure 6:**
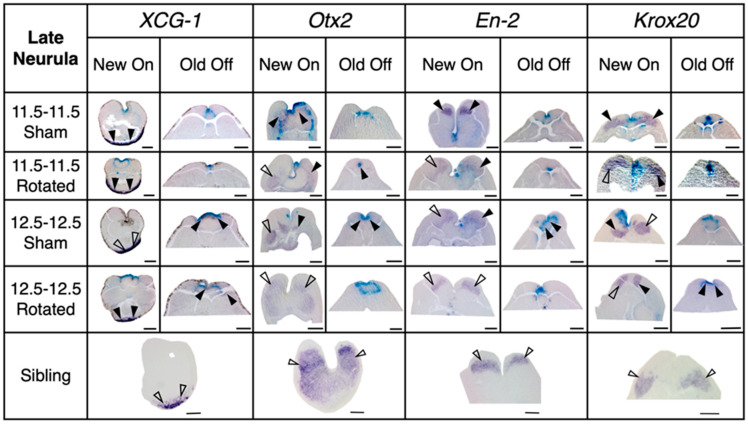
Representative histology for transplants at late neurula stage with sibling control comparisons. Dorsal is facing up. The four regional marker genes *XCG-1*, *Otx2*, *En-2*, and *Krox20* are expressed at the cement gland, eye and forebrain, midbrain, and rhombomeres 3 and 5 of the hindbrain, respectively. Purple stain marked with a solid arrowhead indicates gene expression colocalized with transplant tissue, purple stain marked with an empty arrowhead indicates endogenous gene expression, and blue stain indicates transplant incorporation. Late gastrula rotated embryos show decreased level and more restricted area of marker gene expression at correct locations compared with mid-gastrula sham and rotated embryos. Scale bar represents 250 µm.

**Figure 7:**
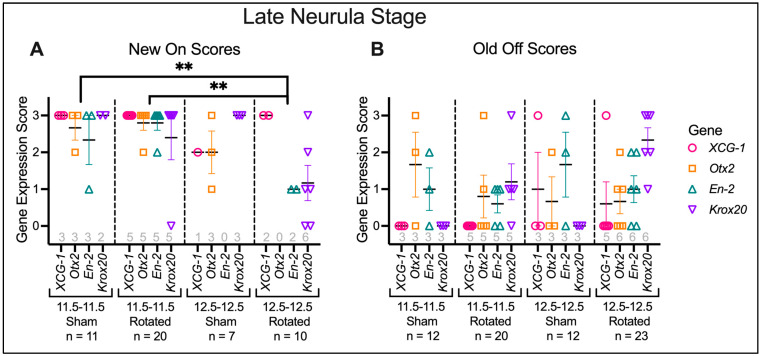
Histology scores for transplants at late neurula stage. Histological sections were scored for expression levels of regional marker genes, both correct “New On” expression (**A**) and ectopic “Old Off” expression (**B**). Horizontal black lines represent the mean, and error bars represent the SEM. ** *p* < 0.01. N for each gene and condition is indicated along the x-axis in gray. Late gastrula rotated embryos have significantly lower levels of correct marker gene expression than mid-gastrula sham embryos (*p* = 0.0077) and mid-gastrula rotated embryos (*p* = 0.0018).

Embryos were assessed for regional marker gene expression at the late neurula stage (St. 18) (Figure 5, Figure 6 and Figure 7). A two-way ANOVA was performed to analyze the effect of transplant stage (mid-gastrula vs. late gastrula) and transplant type (sham vs. rotated) on regional marker gene expression. For “New On” scores, this analysis revealed that the interaction between transplant stage and transplant type was trending towards significance (F (1, 44) = 3.442, *p* = 0.0703). The main effect of transplant stage was significant (F (1, 44) = 9.122, *p* = 0.0042), and the main effect of transplant type was trending towards significance (F (1, 44) = 3.121, *p* = 0.0842). These results indicate that at the late neurula stage, embryos have a decreased ability to correctly bring up regional marker gene expression following late gastrula transplantation and suggest that rotation of the presumptive neural ectoderm worsens this effect. A Tukey post hoc test revealed that late gastrula rotated embryos had significantly lower scores for “New On” than mid-gastrula sham embryos (*p* = 0.0077) and mid-gastrula rotated embryos (*p* = 0.0018). Late gastrula rotated embryos also had lower “New On” scores than late gastrula sham embryos, although this difference was not statistically significant (*p* = 0.1192). No other between-group comparisons were statistically significant. These results indicate that at late neurula stages, late gastrula rotated embryos have a decreased ability to correctly bring up regional marker gene expression, indicating that they are not able to successfully re-pattern their neural axis following inversion at late gastrula stages. Mid-gastrula rotated transplants did not have significantly different “New On” scores compared to sham transplants (*p* = 0.9999), suggesting that embryos are able to correctly regulate expression of regional marker genes following neural axis inversion at mid-gastrula stages.

A two-way ANOVA for “Old Off” scores found no significant main effect of transplant stage (F (1, 63) = 1.516, *p* = 0.2228) or transplant type (F (1, 63) = 0.3335, *p* = 0.5657), or interaction between the two factors (F (1, 63) = 0.4057, *p* = 0.5265), indicating that the amount of ectopic regional marker gene expression did not significantly differ among treatment groups. However, a separate two-way ANOVA analysis on each gene revealed a significant main effect of transplant type on *Krox20* expression (F (1,13) = 19.31, *p* = 0.0007). In this analysis the transplant stage was not significant (F (1,13) = 1.987, *p* = 0.1821), nor was the interaction between the two factors (F (1,13) = 1.987, *p* = 0.1821). These results indicate that rotated embryos showed increased ectopic *Krox20* expression compared to sham embryos, regardless of whether the transplant was performed at the mid- or late gastrula stage. Thus, rotated embryos are not able to fully regulate ectopic gene expression induced by transplantation by the late neurula stage.

#### 3.3.3. Transplants Assayed at Hatching Stages

**Figure 8:**
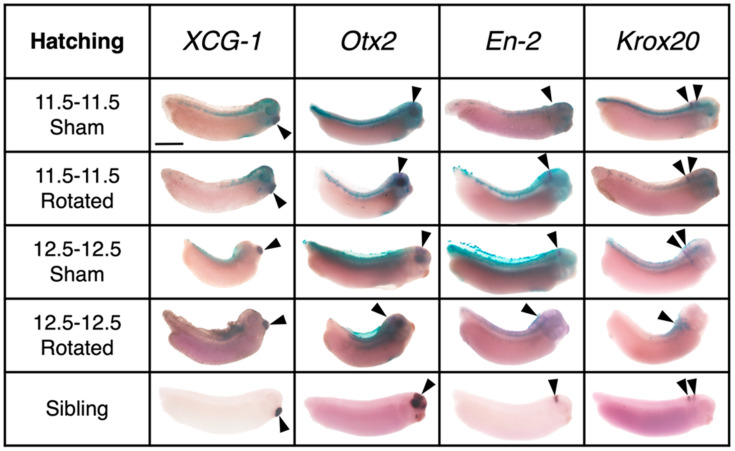
Representative examples of expression of regional marker genes in transplants and sibling controls at hatching stage. Lateral view of all embryos with dorsal facing up; anterior is to the right. Purple stain marked with a solid arrowhead indicates gene expression and blue stain indicates transplant incorporation. Scale bar represents 1 mm.

**Figure 9:**
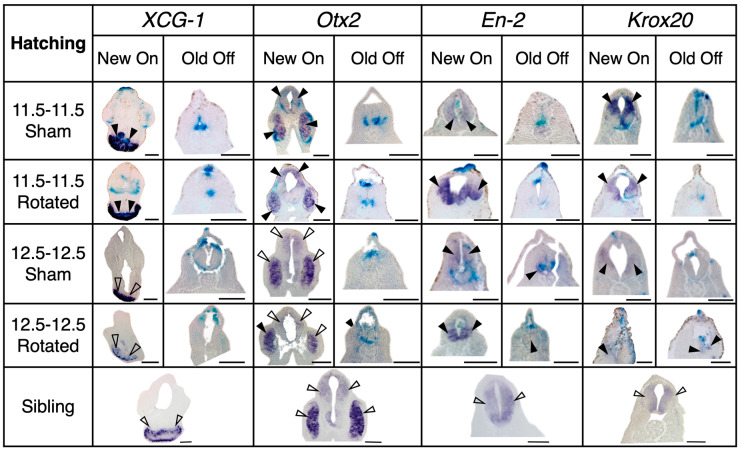
Representative histology for transplants at hatching stage, with sibling control comparisons. Dorsal is facing up. Purple stain marked with a solid arrowhead indicates gene expression colocalized with transplant tissue, purple stain marked with an empty arrowhead indicates endogenous gene expression, and blue stain indicates transplant incorporation. Late gastrula rotated embryos show decreased levels of and more restricted area of marker gene expression at correct places compared with mid-gastrula sham and rotated embryos. Late gastrula rotated embryos also present more ectopic gene expression than mid-gastrula rotated embryos. Scale bar represents 250 µm.

**Figure 10:**
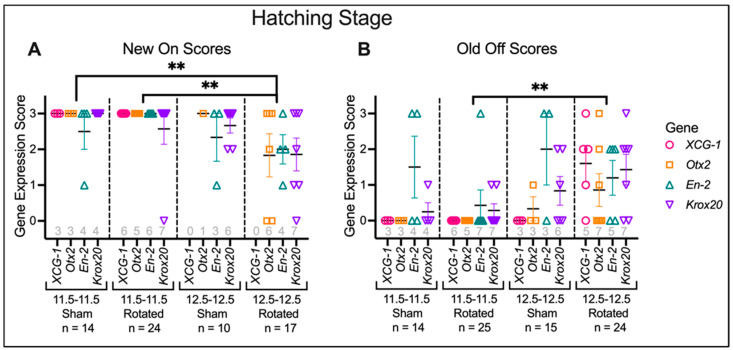
Histology scores for transplants at hatching stage. Histological sections were scored for expression levels of regional marker genes, both correct “New On” expression (**A**) and ectopic “Old Off” expression (**B**). Horizontal black lines represent the mean, and error bars represent the SEM. ** *p* < 0.01. N for each gene and condition is indicated along the x-axis in gray. Late gastrula rotated embryos have significantly less appropriate marker gene expression than mid-gastrula sham (*p* = 0.0064) and Rotated embryos (*p* = 0.0012). Late gastrula rotated embryos also have significantly more ectopic marker gene expression (*p* = 0.0022) than mid-gastrula rotated embryos.

Regional marker gene expression was also assessed for embryos raised to the hatching stage (St. 30) (Figure 8, Figure 9 and Figure 10). A two-way ANOVA was performed to analyze the effect of transplant stage (Mid-Gastrula vs. Late Gastrula) and transplant type (Sham vs. Rotated) on marker gene expression. For “New On” scores, this analysis revealed that the interaction between transplant stage and type was trending towards significance (F (1, 61) = 3.152, *p* = 0.0808). The main effect of the transplant stage was significant (F (1, 61) = 9.100, *p* = 0.0037), and the main effect of transplant type was trending towards significance (F (1, 61) = 2.853, *p* = 0.0963). These results indicate that at the hatching stage, embryos have a decreased ability to correctly bring up regional marker gene expression following late gastrula transplantation and suggest that rotation of the presumptive neural ectoderm worsens this effect. A Tukey post hoc test indicated that the same pairwise differences as found at late neurula stages were present at hatching stages. Late gastrula rotated embryos had significantly lower scores for “New On” than mid-gastrula sham embryos (*p* = 0.0064) and Mid-gastrula rotated embryos (*p* = 0.0012). Late gastrula rotated embryos also had lower “New On” scores than late gastrula sham embryos, although this difference was not statistically significant (*p* = 0.1173). No other between-group comparisons were significant. Late gastrula rotated embryos persist in their inability to correctly express regional marker genes as development proceeds; they do not show compensation for the perturbation over time.

A two-way ANOVA for “Old Off” scores revealed that the main effect of transplant stage was significant (F (1, 74) = 8.476, *p* = 0.0048), while the main effect of transplant type was not significant (F (1, 74) = 0.1046, *p* = 0.7472), nor was the interaction between the two factors (F (1, 74) = 2.616, *p* = 0.1100). A Tukey post hoc test indicated that the only significant pairwise difference was between late gastrula rotated and mid-gastrula rotated embryos, was that late gastrula rotated embryos having significantly higher “Old Off” scores (*p* = 0.0022).

A separate two-way ANOVA analysis on each gene revealed significant effects for *XCG-1* and *Krox20* expression specifically. For *XCG-1* expression, there is a significant main effect of transplant stage (F (1, 13) = 6.194, *p* = 0.0272) and transplant type (F (1, 13) = 6.194, *p* = 0.0272), and a significant interaction between the two factors (F (1, 13) = 6.194, *p* = 0.0272). A post hoc Tukey test indicates that late gastrula rotated embryos have significantly increased ectopic *XCG-1* expression compared to the other three groups (late gastrula rotated vs. late gastrula sham, *p* = 0.0192; late gastrula rotated vs. mid-gastrula rotated, *p* = 0.0052; late gastrula rotated vs. mid-gastrula sham, *p* = 0.0192). For *Krox20* expression, there is a significant main effect of transplant stage (F (1, 20) = 5.762, *p* = 0.0262), suggesting that transplantation of neural tissue at the late gastrula stage results in increased ectopic expression compared to mid-gastrula transplantation, regardless of whether the transplant is sham or rotated. Together, these results suggest that in addition to late gastrula rotated embryos not bringing up regional marker gene expression in the correct location, they also tend to have increased ectopic expression. For both “New On” and “Old Off” scores, mid-gastrula rotated embryos are not significantly different from sham transplant embryos (*p* = 0.9999 for “New On,” *p* = 0.8003 for “Old Off”), indicating that they are similarly able to recover following rotation of their anterior- posterior neural axis.

These results are in general agreement with the initial morphological classification of transplant embryos in suggesting that embryos lose the ability to recover from anterior–posterior neural axis rotation by late gastrula stages. Moreover, in terms of gene expression, late gastrula embryos display a diminished ability to regulate following any type of perturbation (sham or rotation). However, embryos still show plasticity following this perturbation if it is performed at mid-gastrula stages with an ability to regulate appropriate gene expression. Thus, the time between stage 11.5 and 12.5 represents a window of neural axis plasticity during which embryos progressively lose the ability to re-pattern the AP axis following inversion.

### 3.4. Mid-Gastrula Rotation Embryos Recover and Display Appropriate Marker Gene Expression by the Mid-Neurula Stage

**Figure 11:**
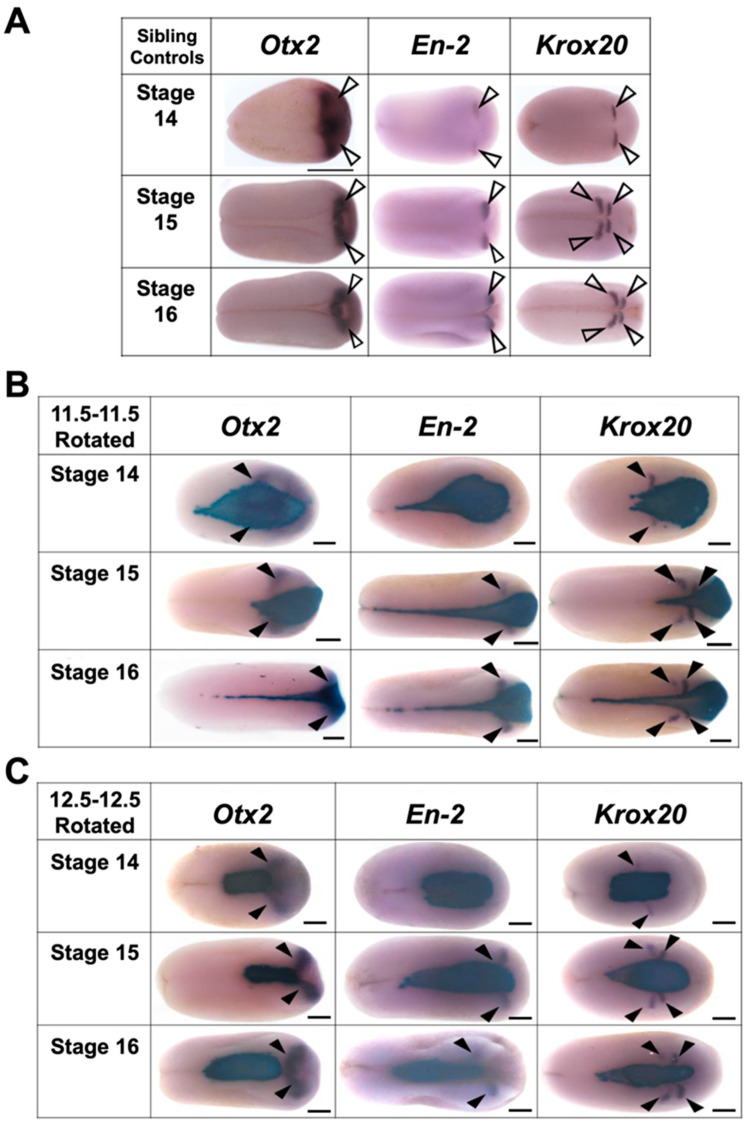
Representative examples of expression of regional marker genes in (**A**) sibling control embryos, (**B**) mid-gastrula rotated transplants, and (**C**) late gastrula rotated embryos at stages 14 to 16. Dorsal view for all embryos, anterior is to the right. Purple stain marked with a solid arrowhead indicates gene expression and blue stain indicates transplant incorporation. Scale bar represents 250 µm.

**Figure 12:**
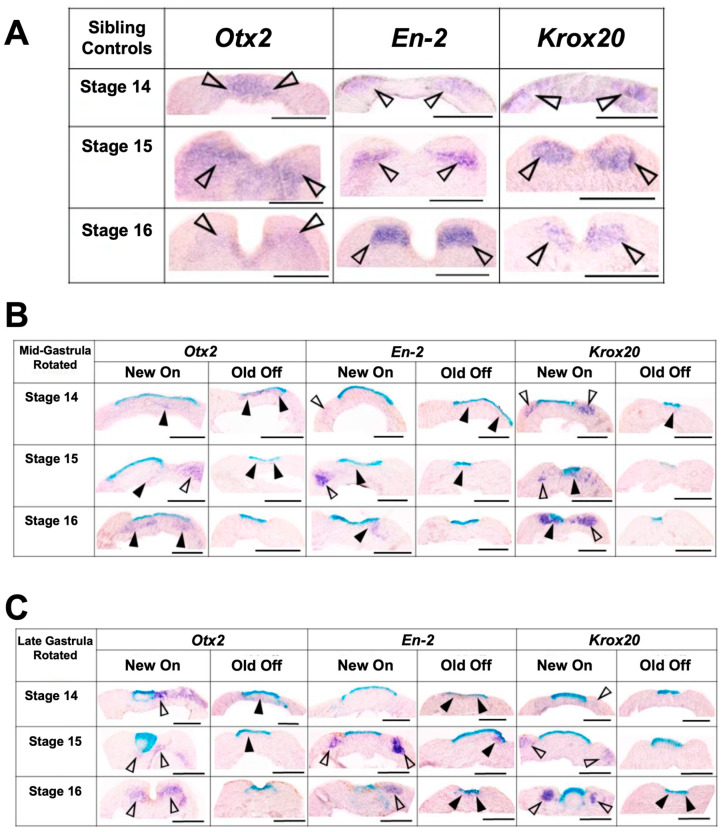
Representative histology images for sibling control embryos (**A**), mid-gastrula rotated transplants (**B**), and late gastrula rotated transplants (**C**) at stages 14–16. Dorsal is facing up. Purple stain marked with a solid arrowhead indicates gene expression colocalized with transplant tissue, purple stain marked with an empty arrowhead indicates endogenous gene expression, and blue stain indicates transplant incorporation. As embryos develop from stage 14–16, sections showed progressive activation of correct marker gene expression and repression of ectopic marker gene expression. Scale bar represents 250 µm.

**Figure 13:**
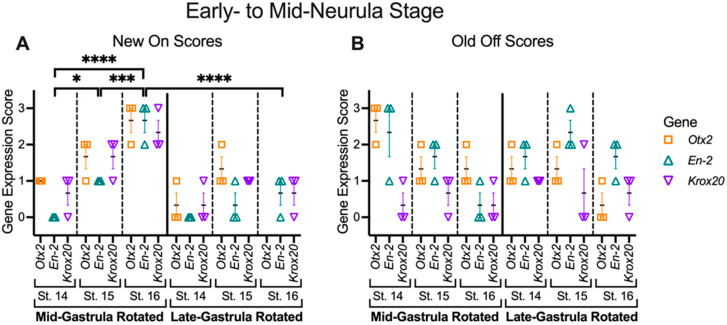
Histology scores for mid- and late gastrula rotated transplants at stage 14 to 16. Histological sections were scored for expression levels of regional marker genes, both correct “New On” expression (**A**) and ectopic “Old Off” expression (**B**). Horizontal black lines represent the mean, and error bars represent the SEM. * *p* < 0.05, *** *p* < 0.001, **** *p* < 0.0001. n = 3 for each stage and gene. For mid-gastrula rotated embryos, stage 15 embryos have significantly higher levels of correct marker gene expression than stage 14 embryos (*p* = 0.0100), and stage 16 embryos have significantly higher levels of expression than both stage 14 (*p* < 0.0001) and stage 15 (*p* = 0.0007) embryos. Stage 16 mid-gastrula rotated embryos also have significantly higher levels of correct marker gene expression than stage 16 late gastrula rotated embryos (*p* < 0.0001).

Given that mid-gastrula rotated embryos had largely recovered and displayed relatively normal marker gene expression by the late neurula stage (St. 18), we wished to determine a more precise time course of their recovery. We therefore performed mid- and late gastrula rotated transplants and fixed the embryos at an early neurula stage (St. 14) and mid-neurula stages (St. 15 and St. 16). Embryos were then assayed for marker gene (*Otx2*, *En-2*, and *Krox20*) expression (Figure 11, Figure 12 and Figure 13). A two-way ANOVA was performed to analyze the effect of rotation stage (mid-gastrula vs. late gastrula) and analysis stage (St. 14, 15, 16) on regional marker gene expression. For “New On” scores, this analysis indicated a significant main effect of rotation stage (F (1, 45) = 38.63, *p* < 0.0001) and analysis stage (F (2, 45) = 22.76, *p* < 0.0001), as well as a significant interaction between the two factors (F (2, 45) = 9.901, *p* = 0.0003). A Tukey post hoc test showed that for all mid-gastrula rotated embryos, those fixed at stage 14, 15, and 16 all had significantly different levels of correct marker gene expression from one another, with older stages having higher expression levels (St. 14 vs. St. 15, *p* = 0.0100; St. 15 vs. St. 16, *p* = 0.0007; St. 14 vs. St. 16, *p* < 0.0001). On the contrary, late gastrula rotated embryos showed no significantly different activation of correct marker genes between any stages, a result that is not surprising given that late gastrula rotated embryos did not recover from the rotation. Expectedly, mid-gastrula rotated embryos also have significantly more correct marker gene expression than late gastrula rotated embryos, but only at stage 16 (*p* < 0.0001). These data indicate that in mid-gastrula rotated embryos, progressive activation of correct marker gene expression in transplant tissues that starts at the early neurula stage is possible (St. 14) and is largely complete by mid-neurula stages (St. 16).

## 4. Discussion

Classic embryological experiments and contemporary molecular investigations have identified the period between early gastrula and neural plate stages as one during which anterior–posterior neural axis patterning and regional identity becomes progressively determined (e.g., [25,28,29,30,31,57,58,59,60]; this was also reviewed in [3,5,22,61]. By the late gastrula stage, the morphogen signaling gradients responsible for AP patterning have already been established [6,62]. These signaling pathways in turn activate their downstream target genes within the neuroectoderm along the AP axis, instructing cells to commit to regionally specific cell fate [3,5,22,61].

While many studies have investigated plasticity within narrow spatial domains of the anterior–posterior axis, for example within the developing hindbrain [43] or neural crest [41,42], less attention has focused on the plasticity of broad regions of the A–P axis during the critical gastrula stages. Many of the earlier studies that did employ larger pieces were of disparate or unknown sizes, and many included varying amounts of underlying mesoderm—all of which made interpretation of the results problematic. The current study addresses the question of anterior–posterior early neural fate plasticity by combining classical embryological techniques and the use of molecular markers to assess the patterning of the AP axis after rotation and transplantation of a relatively large region of standardized size and location of the neural axis without underlying mesoderm [22,23,24,25,26,27,28,29,30]. We identified a narrow window of AP neural axis plasticity between mid- and late gastrula stage, during which period *Xenopus laevis* embryos can recover relatively well from a 180-degree anterior–posterior rotation of their neural axis. Histological analysis of regional marker gene expression along the AP neural axis revealed that embryos whose prospective neural axis was rotated at mid-gastrula stages generally showed appropriate expression of the regional marker genes and minimal ectopic expression of these genes when assayed at late neurula stages and beyond. On the other hand, not only do embryos whose neural axis was rotated at late gastrula stages have statistically significantly less marker gene expression at appropriate locations than those rotated at mid-gastrula stages, these embryos also have significantly more ectopic marker gene expression. The results show that *Xenopus laevis* embryos lose their ability to activate correct regional marker gene expression and turn off ectopic marker gene expression following AP neural axis rotation as they develop from the mid- to late gastrula stage. Moreover, the recovery from AP neural axis rotation begins by neural plate stages (St. 14) and is very significant by mid-neurula stages (St. 16), with recovery progressively continuing through later stages. Embryos with a mid-gastrula rotated transplant showed less inappropriate, ectopic expression when assayed at later hatching stage compared to those assayed at late neurula stages. This difference was not observed in embryos with rotated transplants performed at late gastrula stages. The poor level of correct and ectopic marker gene expression in transplant tissues remained constant throughout the developmental stages we examined.

These findings suggest a relatively narrow window of broad AP neural axis plasticity that is present at mid-gastrula stage but has closed by late gastrula stage. These results are consistent with earlier studies that revealed a labile period for AP neural axis re-patterning ability between mid- and late gastrula stage [57,58]. They also consistent with findings of Szaro et al. [29] who transplanted posterior ectoderm into the presumptive eye region at neural plate stages and observed mixed degrees of regulation, with very few transplants showing normal regulated development.

Our findings are interpretable in light of the updated “activation-transformation” model of neural patterning (reviewed in [63,64]). According to the initial hypothesis, the entire neuroectoderm is first “activated” to an anterior fate during neural induction, and the posterior region is subsequently “transformed” into more caudal neural fates [65,66,67,68]. Inhibition of the BMP and Wnt signaling pathways were shown to produce initial anterior fates with FGF, Wnt, and retinoic acid acting as posteriorizing, transforming signals [10,68,69]. In order to resolve a significant number of inconsistencies with this initial model, including the presence of ongoing induction of tail CNS from neuromesodermal precursors (NMPs) that never appear to adopt an anterior fate, an updated model has been proposed which argues for two distinct signaling centers and that “primary regionalization” distinguishing the spinal cord from the rest of the CNS is established prior to neural induction [8,9]. While the activation-transformation framework applies well for the hindbrain, midbrain and forebrain, with low BMP and Wnt acting as a transforming factor for hindbrain fates, the spinal cord region is induced and patterned separately, independently, and progressively from a population of NMPs. Mechanistically this involves different roles for these signaling pathways than for the more anterior part of the nervous system, with the BMP signaling promoting spinal cord fates via activation of FGF signaling, with Wnt signaling presently at low levels [3,9,70]. This is consistent with evidence showing the dynamic and changing nature of Wnt signaling throughout neural development and the importance of timing and duration of Wnt and BMP signaling in cell fate decisions [3,69,71]).

The window of AP neural axis plasticity we observed between mid- and late gastrula stages, likely representing the period between activation and transformation of the hindbrain regions. As the posterior presumptive neuroectoderm is being transformed by the caudalizing signal to a posterior neural fate between the mid- and late gastrula stage, more and more cells along the AP neural axis become committed to their regional cell fate, and AP neural axis plasticity diminishes. The presence of cells from the spinal cord regions still undergoing neural induction and regionalization may allow for mid-gastrula plasticity and repurposing of the Wnt and FGF signaling pathways in rotated tissue. However, we do observe that the more posterior a marker gene is expressed (e.g., *Krox* in our experiments), the more dysregulated it is in all categories of transplants, consistent with further distance from the transforming signal.

The regulation we observe in embryos with mid-gastrula rotated transplants may also be enhanced by the significant cross-talk among the Wnt, BMP and FGF pathways [69,72], including genes such as Zbtb14 that inhibits BMP signaling and promotes Wnt signaling. Expressed through neurula stages in anterior tissues, even though it promotes posteriorization, [73]), it was suggested that Zbtb14 only enhances Wnt signaling if Wnt ligand are present, which they are when tissue is rotated, thus allowing for regulation of the rotated tissue transplant. Virgirinia et al. [74] have shown that Cdc-2-like kinase 2 (Clk2) not only promotes overall neural development but also elicits the expression of both anterior and posterior neural genes via BMP inhibition and FGF pathway activation; given that Clk2 is expressed at transplant stages, this may be a mediator of re-patterning. Reports of considerable heterogeneity of gene expression that controls AP patterning may also contribute to plasticity in patterning [59] as would the robustness displayed by key signaling systems such as retinoic acid [75]. Girgin et al. [76] showed that inhibition of Wnt signaling gastruloids generated using activin A and FGF2 resulted in significant degree of anterior–posterior patterning and suggested that there may be significant compensatory mechanisms for generating a patterned axis, a feature that also bestows enhanced early plasticity.

Epigenetic modifiers are known to play a key role in regulating plasticity [3,8,77,78,79,80]. The gradual restriction of expression of such factors to the dorsal midline during the late gastrula stage further corroborates our proposed window of AP neural plasticity that allows mid-gastrula rotated tissue to respond to new signals from underlying surrounding tissues to adopt new fates, while later gastrula tissues have irreversibly committed to a certain fate. As for the continued recovery between late neurula and hatching stage, this could be due to a different mechanism from the initial compensation like secondary neurogenesis [81], but its exact nature requires further investigation. Both a loss in inducing signal from the underlying mesoderm and the loss of competency of the overlying ectoderm (or signals within the ectoderm) could contribute to the loss of neural axis plasticity as embryos develop from mid- to late gastrula stage. That both germ layers play a key role in plasticity is not surprising, given that both the mesoderm and neuroectoderm have been shown to be essential for neural induction and AP patterning of the neural axis [4,64,82]. Previous studies have found the age of the transplant from the donor to be the deciding factor in the amount of induced neural tissue, with older transplants producing less neural tissue [83]. However, these experiments did not involve the rotation of AP neural axis and thus did not address the question of AP neural axis plasticity. Servetnick and Grainger [84] have shown that ectodermal neural competence results from an autonomous developmental timer within the ectoderm itself—one which contributes to allowing the ectoderm to continue to respond at early gastrula stage but significantly declines by later gastrula stages. On a molecular level, Mancini et al. [85] have shown the importance of planar signals emanating from the blastopore and specifying PCP progressively through the neuroectoderm.

While classic well studied signaling systems are likely mediating the ability of the mid-gastrula rotated transplants to adapt to their new environment, additional mechanisms may also be involved. Ion fluxes have been shown to regulate neural patterning [86] and regeneration and Levin’s group have identified long-range signaling neurotransmitter and electrical signaling as important for patterning and regeneration [87].

Recently there has been insight on the importance of mechanical forces and the ongoing crosstalk between these physical signals and altered gene expression [60,88]. Recent findings showing how chromatin responds to cellular deformation suggests that mechanical stress may play a role in plasticity [89]. Additional players include HIF1alpha, which is known to act in conjunction with Wnt signaling and act upstream of injury-induced gene expression responses and is developmentally regulated [90]. Indeed, given that our data suggest that the ability to restore appropriate gene expression following any type of perturbation (Sham or Rotation transplant) is diminished by late gastrula stages, developmentally regulated responses to injury may be playing an important role.

In this study, we combined the classical embryological approach of rotation transplantation and newer molecular techniques to investigate the timing, extent, and contributing tissues of AP neural axis plasticity in *Xenopus laevis*. In conclusion, this research identified a narrow window of profound anterior–posterior neural axis plasticity between the mid- and late gastrula stage in *Xenopus laevis* embryos. We were able to show that the progressive recovery from AP neural axis rotation commences at the neural plate stage and is largely complete by the mid-neurula stage, although recovery continues later in development. Despite the ability to draw these conclusions, this study has certain limitations. Transplants were performed at only two stages and with a single size and location of neural tissue. Resulting phenotypes were analyzed at hatching stages and not a later stage of development. The four genes were used as regional marker genes, which in and of themselves could not elucidate the underlying mechanisms of this plasticity. Nevertheless, this study provides the foundation for further investigations that could assay gene expression and tissue lineage at the single-cell level as well as for experiments designed to test physiological function of perturbed embryos at later developmental stages. Additionally, further investigations, including a complete transcriptome analysis, will reveal the precise molecular mechanisms of this profound plasticity and the relative roles of the dynamic signaling pathways, transcriptional targets, epigenetic factors, and mechanical stresses.

## Data Availability

All data is available upon reqeust.
